# Genome-wide identification of novel ovarian-predominant miRNAs: new insights from the medaka (*Oryzias latipes*)

**DOI:** 10.1038/srep40241

**Published:** 2017-01-10

**Authors:** Amine Bouchareb, Aurélie Le Cam, Jérôme Montfort, Stéphanie Gay, Thaovi Nguyen, Julien Bobe, Violette Thermes

**Affiliations:** 1 LPGP, INRA, 35000 Rennes, France

## Abstract

MicroRNAs (miRNAs) are small, highly conserved non-coding RNAs that play important roles in the regulation of many physiological processes. However, the role of miRNAs in vertebrate oocyte formation (*i.e.,* oogenesis) remains poorly investigated. To gain new insights into the roles of miRNAs in oogenesis, we searched for ovarian-predominant miRNAs. Using a microarray displaying 3,800 distinct miRNAs originating from different vertebrate species, we identified 66 miRNAs that are expressed predominantly in the ovary. Of the miRNAs exhibiting the highest overabundance in the ovary, 20 were selected for further analysis. Using a combination of QPCR and *in silico* analyses, we identified 8 novel miRNAs that are predominantly expressed in the ovary, including 2 miRNAs (miR-4785 and miR-6352) that exhibit strict ovarian expression. Of these 8 miRNAs, 7 were previously uncharacterized in fish. The strict ovarian expression of miR-4785 and miR-6352 suggests an important role in oogenesis and/or early development, possibly involving a maternal effect. Together, these results indicate that, similar to protein-coding genes, a significant number of ovarian-predominant miRNA genes are found in fish.

Oogenesis and early embryogenesis are highly regulated and coordinated biological processes requiring a large network of gene interactions and tightly regulated genes. During oogenesis, maternal transcripts and proteins are produced and accumulated in oocytes to prepare for meiosis resumption and early embryo development^1^. Genes that exhibit an oocyte-predominant expression pattern have important functions during oogenesis and early embryogenesis^2^,^3^,^4^. These genes are also called oocyte-specific genes^2^.

MicroRNAs (miRNAs) are small non-coding RNAs (19–24 nt) that modulate gene expression at the post-transcriptional level by repressing mRNA translation or by regulating mRNA degradation. Most miRNAs are evolutionarily conserved and play important roles in many physiological and pathological processes^5^,^6^,^7^. The contribution of miRNAs to oogenesis and early embryo development was demonstrated in mice with the report of an arrest of zygotic development after the loss of specific maternal miRNAs and infertility in Dicer1-deficient females^8^,^9^. It was also shown that a specific miRNA, miR-430, is responsible for maternal mRNA clearance during the embryonic development of zebrafish^10^. Many studies have reviewed the critical roles played by miRNAs in controlling the expression of genes essential for folliculogenesis and early embryogenesis^11^,^12^,^13^,^14^. Significant efforts have been made to identify ovarian miRNAs using microarrays or high-throughput sequencing^15^,^16^,^17^,^18^,^19^. However, no comprehensive overview on the ovarian miRNome is available. Additionally, data on ovarian-predominant miRNAs and on the roles of individual miRNAs during oogenesis and early embryonic development remain scarce, especially in non-mammalian models. Based on the hypothesis that ovarian-predominant miRNA genes would also play important roles in oogenesis and/or in early development similar to protein-coding genes, we searched for ovarian-predominant miRNAs in medaka (*Oryzias latipes*). To this end, we designed a miRNA microarray displaying most of the vertebrate miRNAs available in miRBase. We used this microarray to analyze the expression of miRNAs in 10 different tissues. Here, we report the identification of 66 miRNAs that are significantly overabundant in the ovary in comparison to other tissues. Using a combination of QPCR and *in silico* analyses, we identified 8 miRNAs that are predominantly expressed in the ovary, including 2 miRNAs that exhibit strict ovarian expression. Of these 8 miRNAs, 7 were previously uncharacterized in fish. Together, these results indicate that a significant number of ovarian-predominant miRNA genes are found in fish, similarly to protein-coding genes. The strict ovarian expression of miR-4785 and miR-6352 suggests important roles in oogenesis or early development, possibly involving maternal effects. Our findings provide new insights into the participation of ovarian-specific miRNAs in oogenesis and/or early embryo development.

## Results

### Identification of novel ovarian-predominant miRNAs using microarray

**Figure 1:**
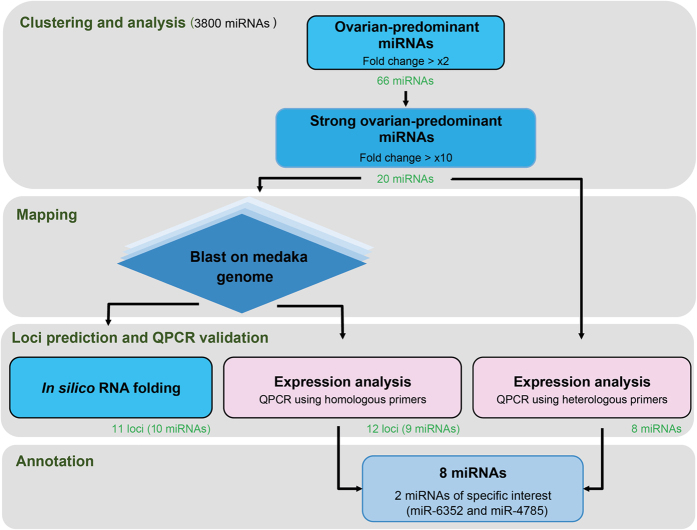
Workflow used to analyze the data and identify novel ovarian-predominant miRNAs in medaka.

**Figure 2:**
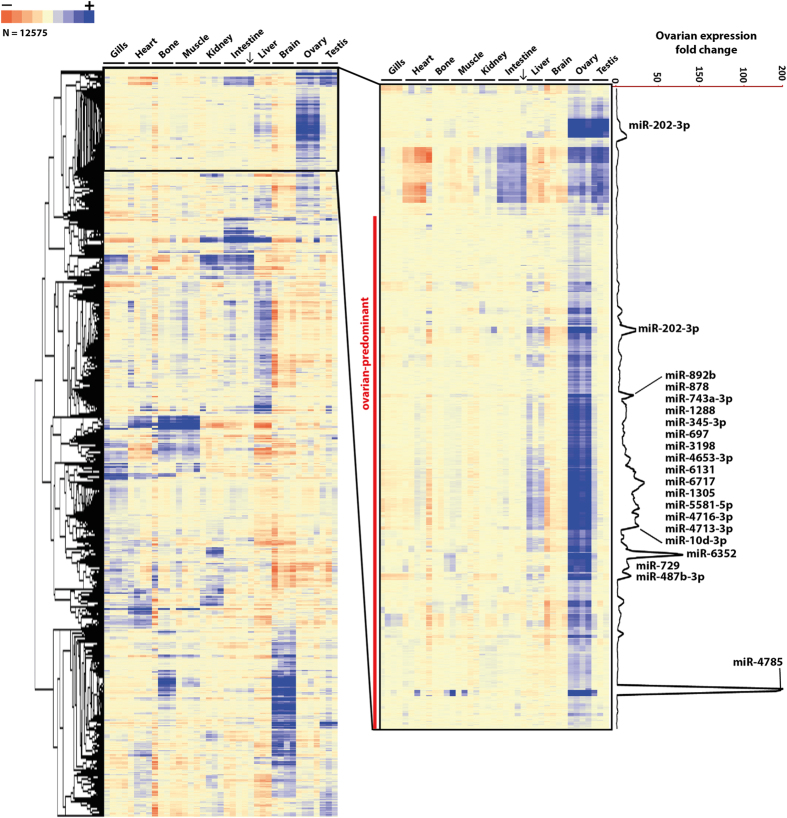
MicroRNA expression profiling in different medaka tissues. Unsupervised clustering analysis of the differentially expressed microRNAs. Each row represents a single probe (16 probes per miRNA) and each column represents a tissue sample (gills, heart, bone, muscle, kidney, intestine, liver, brain, ovary and testis). Data were median-centered prior to the clustering analysis. For each miRNA probe, the expression level within the sample set is indicated using a color density scale. Blue and red are used for overexpression and underexpression, respectively. White is used for median expression. The arrow indicates a testis sample that clusters with the intestine samples, which is probably due to tissue contamination during sampling.

**Figure 3:**
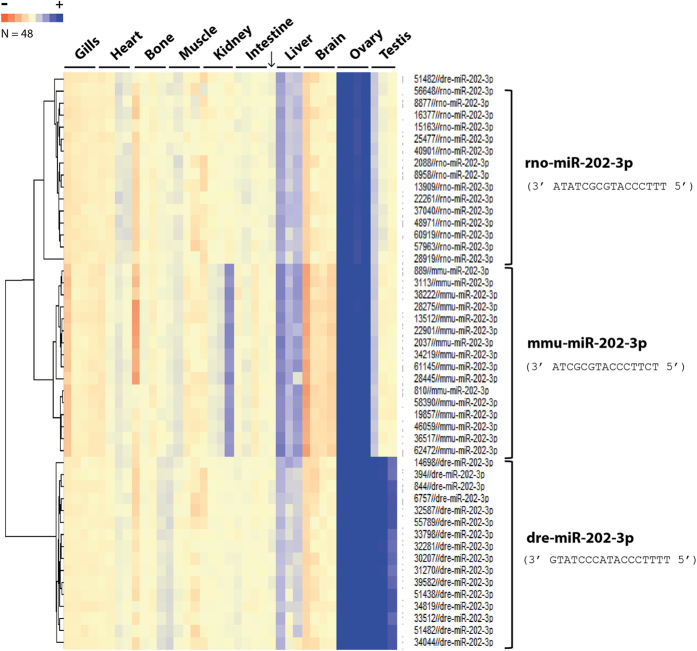
Expression profiles of miR-202-3p in different medaka tissues. Each row represents a single probe (16 probes per miRNA). Each column represents a medaka tissue RNA sample. The zebrafish probes (dre-miR-202-3p) display gonadal-predominant expression profiles, whereas probes from rat and mouse (rno-miR-202-3p and mmu-miR-202-3p, respectively) exhibited strict ovarian-predominant expression profiles. Probe sequences are indicated.

To identify ovarian-predominant miRNAs in medaka, we performed a miRNA microarray experiment using a generic microarray that displayed a large repertoire of vertebrate miRNA sequences as previously described^20^. The workflow applied to analyse the resulting dataset is described in Fig. 1. Of the 3800 miRNAs present on the microarray, 66 miRNAs (corresponding to 71 probes) exhibited a significant ovarian-predominant expression (>2-fold, p < 0.005, Fig. 2, Supplemental Fig. S1). Thirty-four of these miRNAs probes corresponded to human sequences, 16 to mice sequences, 14 to rat sequences, 4 to zebrafish sequences, 2 to chicken sequences and 1 to a medaka sequence. For further analysis, we selected 20 miRNAs displaying dramatic ovarian-predominant expression (>10-fold, p < 0.005). Of these 20 miRNAs was zebrafish miR-202-3p (dre-miR-202-3p), which we selected despite its detection in both the ovary and testis (Fig. 3). Interestingly, the microarray profiles displayed two other probes (rno-miR-202-3p and mmu-miR-202-3p) that exhibited strict ovarian-predominance and suggested the presence of a miR-202 isomiR in medaka.

### Further QPCR validation of the most ovarian-predominant miRNAs

**Figure 4:**
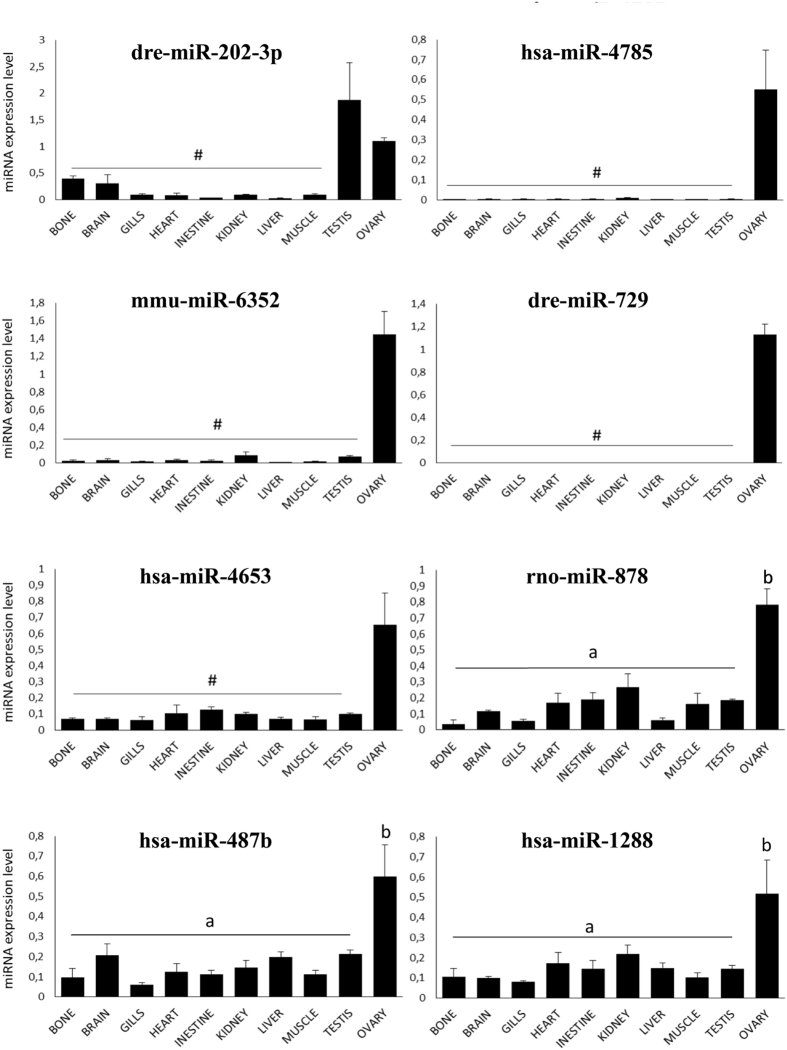
miRNA expression profiles obtained by QPCR using heterologous primers. Four samples from different individual medaka fish were used for each tissue. Expression levels were measured in duplicates and the mean values (±SD) are displayed on the graphs. Data were normalized to the abundance of 18 S. a and b indicate expression levels that are significantly different (Wilcoxon test, p < 0.05). ab, expression levels not significantly different from a and b expression levels not significantly different from the background signal.

For the 20 ovarian-predominant miRNAs, tissue expression was analyzed by QPCR and compared to the profiles obtained in the microarray study. The expression profiles were generated using primers corresponding to heterologous miRNAs (*i.e.,* heterologous primers) originating from various vertebrate species and according to the sequences present on the microarray (Supplemental Fig. S2). In the QPCR analysis, 7 candidates exhibited an ovarian-predominant expression profile that was consistent with the microarray data (hsa-miR-4785, mmu-miR-6352, dre-miR-729, hsa-miR-4653, rno-miR-878, hsa-miR-487b and hsa-miR-1288); excepted dre-miR-729, these miRNAs were previously uncharacterized in fish (Fig. 4). The ovarian-predominant expression was especially strict for hsa-miR-4785, mmu-miR-6352, dre-miR-729 and hsa-miR-4653. It was less pronounced though still significant for rno-miR-878, hsa-miR-487b and hsa-miR-1288. The expression profile of dre-miR-202-3p revealed a strong and significant overabundance in the testis and ovary compared to other tissues. In contrast, 3 of the 20 miRNAs (mmu-miR-743a-3p, hsa-miR-4713-3p and hsa-miR-4716-3p) were expressed in all tissues at different levels without any overexpression in the ovary. For the other 8 candidates (hsa-miR-5581-5p, hsa-miR-3198, hsa-miR-6131, hsa-miR-6717-5p, rno-miR-345-3p, mmu-miR-697, hsa-miR-892b and dre-miR-10d-3p), QPCR resulted in more than one product, which made it impossible to conclude on their overexpression in the ovary. Finally, we failed at amplifying hsa-miR-1305 by QPCR.

### Identification of cognate medaka miRNA genes

To identify the cognate medaka miRNA sequences, we searched the medaka genome for miRNA genes that were orthologous to the miRNA sequences identified in the microarray analysis. For the 20 ovarian-predominant miRNAs candidates, corresponding sequences were aligned (BLAST) on the medaka genome. All 20 miRNAs had at least one match in the medaka genome with at least 90% identity over 86% of the query length. The best hits (from 2 to 4 depending on the candidate) were considered as potential loci for being the corresponding medaka miRNA gene(s) (Table 1). The surrounding genomic regions were used as templates to determine the potential whole precursor sequences and the secondary structures were computed for these sequences. *In silico* prediction of the hairpin structures supported the identification of 11 loci that encode for 10 miRNAs (Supplemental Fig. S3; Table 1). Among them, 3 are miRtrons (mir-6352, mir-1305 and mir-6717) that are present in the introns of ENSORLG00000017404, *fam171a1* (family with sequence similarity 171, member A1) and *shank1* (SH3 and multiple ankyrin repeat domains 1), respectively. One is in an exon in the protein-coding transcript of the *rasip1* gene (ras interacting protein 1), while 9 are intergenic miRNAs.

### Expression profiles of the mature medaka miRNAs

**Figure 5:**
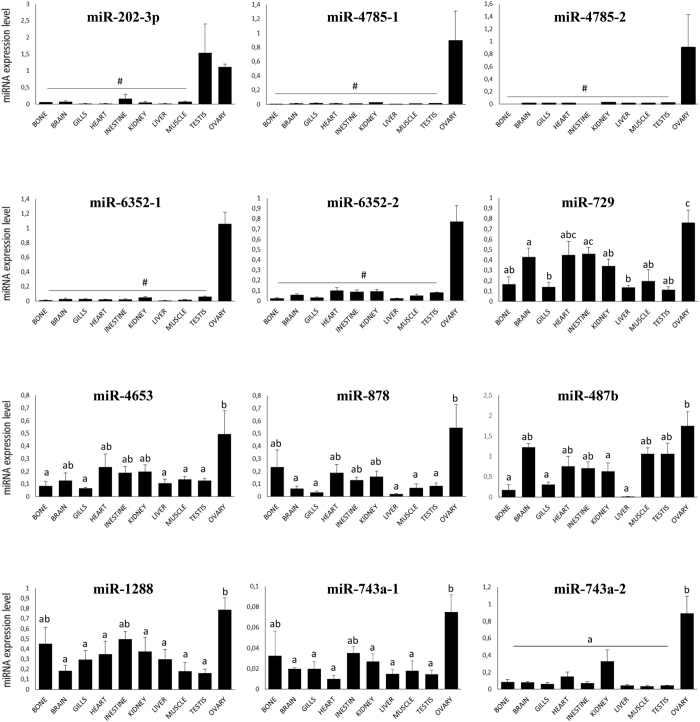
miRNAs expression profiles obtained by QPCR using medaka homologous primers. Four samples from different medaka fish were used for each tissue. Expression levels were measured in duplicates and the mean values (±SD) are displayed on the graphs. Data were normalized to the abundance of 18S. a and b indicate expression levels that are significantly different (Wilcoxon test, p < 0.05). ab, expression levels not significantly different from a and b. #expression levels not significantly different from the background signal.

For each medaka genomic locus predicted for the 20 candidate miRNAs, we analyzed the expression profile of the corresponding mature miRNAs to confirm the ovarian-predominant expression previously obtained by the microarray analysis or by heterologous QPCR. For each locus, the corresponding medaka primer (*i.e.,* homologous primer) was designed for QPCR (Fig. 5 and Supplemental Fig. S4). MiR-202-3p exhibited a strong expression in both the ovary and testis compared with other tissues. Four loci exhibited strict ovarian-predominant expression of the mature sequences, including miR-4785-1, miR-4785-2, miR-6352-1 and miR-6352-2. Significant ovarian-predominant expression was also observed for miR-743a-2, even though it was detected in all tissues, especially the kidney. For the other loci (miR-729, miR-4653, miR-878, miR-487b, miR-1288 and miR-743a-1), overexpression in the ovary was not systematically significant when compared with other tissues. These 12 loci corresponded to 9 different miRNAs (Table 1). Three of them exhibited an ovarian–predominant expression profile (miR-4785, miR-6352 and miR-743a-2).

### Target prediction

MiR-4785 and miR-6352 were strictly ovarian-predominant when analyzed by QPCR using homologous or heterologous primers. To identify cellular processes putatively targeted by these 2 miRNAs, target genes were predicted using TargetScan and Miranda (Supplemental Fig. S6). A total of 11 and 17 common genes were identified for miR-4785 and miR-6352, respectively. The expression profiles of some putative targets were also examined in medaka using the PhyloFish tissue repertoire database (http://phylofish.sigenae.org)^21^, with a special interest for genes expressed in ovary (Supplemental Fig. S6).

## Discussion

Ovarian-predominant genes are known to play important roles in folliculogenesis, oocyte growth and maturation throughout oogenesis and in early embryo development^22^,^23^,^24^. While most studies have been conducted in mammals, studies in fish have shown that many oocyte-specific genes could also be found in fish^25^,^26^. Oocyte-specific features are typical of maternal-effect genes and play key roles in early embryonic development. For instance, Nucleoplasmin (*Npm2*) is a maternal-effect gene required for embryonic development beyond zygotic genome activation (ZGA) in both mice and zebrafish^3^,^27^. Along with other evidence, this suggests that oocyte-specific genes would be conserved in vertebrates both in terms of function and expression pattern (*i.e.,* oocyte-specific expression). Therefore, our working hypothesis was that ovarian-predominant miRNA genes would also play important roles in oogenesis and early embryonic development in a similar manner to protein-coding genes. In the present study, we were able to identify 66 miRNAs exhibiting ovarian-predominant expression. Of the 66 ovarian-predominant miRNAs selected from the microarray data, 10 miRNAs (including mmu-miR-743a, rno-miR-878 and hsa-miR-487b) were previously identified in the mouse ovary or germline^28^. Two miRNAs (hhi-miR-145 and hhi-miR-202) were previously shown to be predominantly expressed in gonads compared with non-reproductive organs in vertebrates^20^,^29^,^30^,^31^. Finally, 54 miRNAs (including mmu-miR-6352, hsa-miR-4785, hsa-miR-1305, and hsa-miR-5581) had never been reported as ovarian-predominant miRNAs in any vertebrate species. The expression of some miRNAs observed here in a tissue-dependent manner is consistent with previous results obtained in rainbow trout^32^. Similarly, it was recently demonstrated in humans that the majority of miRNAs was neither specific to single tissues nor housekeeping miRNAs. Nonetheless, these investigators concluded that many different miRNAs and miRNA families were predominantly expressed in certain tissues^33^. To our knowledge, no study has ever specifically aimed at identifying ovarian-predominant miRNAs in non-mammalian vertebrates. Therefore, we report here, for the first time in any vertebrate species, more than 60 miRNAs that are significantly and predominantly expressed in the ovary. These results were obtained using a generic microarray displaying mature miRNA sequences originating from various vertebrate species. In general, microarray expression profiles can be confirmed by quantitative QPCR, as discrepancies between the two techniques are limited^34^; further, this methodology was previously used in rainbow trout. In our study, some miRNAs that exhibited ovarian expression profiles in the microarray study were detected in all tissues by QPCR. The presence of different isomiRs could explain, at least in part, these differences in the expression profiles. This is especially true if sequencing originally identified the miRNA sequence present on the microarray. Deep sequencing now allows the detection of variability in miRNA mature sequences, meaning that many different sequences can be generated and subsequently sequenced from the same miRNA precursor. Consistently, previous studies showed that isomiR expression patterns differ between cell lines and/or tissue types^35^,^36^. Sorting and identifying isomiRs remains a challenge in miRNA studies, as the sequence diversity of isomiRs can vary on both ends. This can be an issue for distinguishing mature isoforms from isomiR species^37^,^38^. More generally, being able to connect a murine or human miRNA gene to its corresponding medaka ortholog has limited our study, as is also the case for protein-coding genes^39^. Direct mapping of heterologous miRNA sequences on the medaka genome revealed that sequences could map on either one or several loci. In the present study, we identified 12 medaka miRNA genes that corresponded to 9 mature miRNAs. For instance, mmu-mir-6352, hsa-mir-4785 and mmu-mir-743a each have two co-orthologs in medaka (ola-mir-6352-1 and ola-mir-6352-2, ola-mir-4785-1 and ola-mir-4785-2, and ola-mir-743a-1 and ola-mir-743a-2, respectively) that could possibly result from the teleost genome duplication, while only one copy of ola-mir-202, ola-mir-487b, ola-mir-4653, ola-mir-878 and ola-mir-1288 appear to be present in the medaka genome^40^,^41^. We also identified another co-ortholog for ola-mir-729, even though we have been unable to detect it by QPCR. Because the identification of cognate medaka miRNAs is not always straightforward, we used a combination of evidence including sequence matches on the medaka genome, QPCR expression and miRNA folding prediction. The corresponding information is summarized in Table 1 for the 20 ovarian-predominant miRNAs selected. The folding information should be used with caution, as all miRNAs do not necessarily exhibit this feature^42^. Finally, it should be stressed that despite the overall conservation of the ovarian-predominant patterns, we observed some differences between the QPCR profiles obtained using homologous and heterologous primers. For miR-729, miR-4653, miR-878, mir-487b, miR-1288 and miR-743a-1, ovarian overexpression was not always significant, depending on the tissue, when QPCR was performed using homologous primers. This can probably be explained by the difficulty in designing primers without information on the exact mature miRNA sequence in medaka.

Among the miRNAs exhibiting strong expression in the ovary, miR-202 is of special interest. MiR-202 was previously shown to be expressed in the gonads of Atlantic halibut, rainbow trout, frog, chicken, and mice^20^,^29^,^30^,^43^. Moreover, sexually dimorphic expression of miR-202 was reported to occurs during gonadal development in both chicken and mouse, with predominant expression of the miRNA in the testis^29^,^30^. In agreement with existing data, we report here a strong overexpression of miR-202 in the medaka ovary and testis compared with non-reproductive tissues. This was further confirmed by QPCR using both zebrafish and medaka primers. Further analysis of our microarray revealed two probes from rat and mouse that exhibited strict ovarian-predominance. This suggests that miR-202-3p in medaka has an isomiR with a strict ovarian expression. Further characterization by small-RNA sequencing is required to determine its sequence and precisely analyze its function in gonads.

In addition, we report here 8 novel ovarian-predominant miRNAs (miR-4785, miR-6352, miR-4653, miR-878, miR-487, miR-1288, miR-743, miR-729) that were either never previously described in fish and/or never shown to be involved in oogenesis in any animal species. The ovarian-predominant expression of these miRNAs was thus previously unsuspected. Dre-miR-729 was characterized in medaka, where it was found in photoreceptor cells^44^. Rno-miR-878 is downregulated in rat liver fibrotic tissues^45^. Hsa-miR-487b is more widely expressed in endothelial and muscle cells in humans and mice, including in cancer cells^46^,^47^,^48^. Hsa-miR-1288 is upregulated in ectopic pregnancy patients^49^ and associated with the progression of colorectal cancer^50^. Mmu-miR-743a is expressed in mouse brain and testis and has possible roles in oxidative stress and neurodegeneration^51^,^52^. Hsa-miR-4785 is downregulated in human nasal epithelial cells treated with the TLR3 ligand poly(I:C)^53^.

For the 2 miRNAs (miR-4785 and miR-6352) that exhibited a strict ovarian-predominant expression, we predicted mRNA targets to identify potential cellular functions (Supplemental Fig. S6). Among the predicted targets of miR-4785 was *fshr*, a gene playing a major role in the ovary through its role in mediating Fsh (Follicle stimulating hormone) action on oogenesis in vertebrates. In zebrafish, disruption of *fshr* leads to a complete failure of follicle recruitment into vitellogenesis with all ovarian follicles arrested at the primary growth-previtellogenic transition^54^. Similarly, we analyzed the putative target genes of miR-6352 and we predicted the *smg8, ddx20* and *ddx6* genes. These later are all expressed in the ovary, according to the PhyloFish database. *Smg8* encodes for a RNA binding protein that controls the maternal mRNA degradation and zygotic genome activation in the early embryo^55^,^56^. This information is consistent with a possible role of miR-6352 during the early embryonic development. MiR-6352 was also predicted to target *ddx6* and *ddx20* genes that encode transcriptional repressors. While *ddx6 (DEAD-box protein-103*/*gemin3)* is known to regulate the meiosis progression^57^, *ddx20* directly represses the transcriptional activity of the transcription factor *foxL2* and the steroidogenic factor *SF-1*^58^,^59^. *FoxL2* and *SF-1* are both key genes for gonadal differentiation^60^,^61^. These data are consistent with a possible role of miR-6352 in ovarian development and oocyte formation.

## Conclusion

Using microRNA microarrays, we identified 66 novel miRNAs predominantly expressed in medaka ovary. Of the miRNAs exhibiting the highest overabundance in the ovary, 20 were selected for further analysis. Using a combination of QPCR and *in silico* analyses, we identified 8 miRNAs (miR-4785, miR-6352, miR-4653, miR-878, miR-487, miR-1288, miR-743 and miR-729) that are ovarian-predominant, including 2 miRNAs that exhibit strict ovarian expression (miR-4785 and miR-6352). Of these 8 miRNAs, 7 were previously uncharacterized in fish (miR-4785, miR-6352, miR-4653, miR-878, miR-487, miR-1288 and miR-743). These results indicate that a significant number of ovarian-predominant miRNA genes are found in the medaka ovary, similarly to protein-coding genes. The strict ovarian expression of miR-4785 and miR-6352 suggest important roles in oogenesis and/or early development, possibly involving a maternal effect. Together, our findings provide new insights into the participation of ovarian-specific miRNAs in oogenesis and/or early embryonic development. Further analyses, including functional validation and target identification of these ovarian-predominant miRNAs, will be necessary to decipher their roles in the regulation of gene expression during oogenesis and early embryogenesis in fish.

## Methods

### Tissue collection

All experimental procedures were carried out in strict accordance with French and European regulations on animal welfare and were approved by the INRA LPGP Animal Care and Use Committee (no. M-2014-5-VT/M-2014-6-VT). Adult medaka (*Oryzias latipes*) from the CAB strain were raised at 28 °C under a reproduction photoperiod (14 hours light/10 hours dark). We collected samples corresponding to 10 different tissues (gills, heart, bone, muscle, kidney, intestine, liver, brain, ovary and testis) from 4 different females. In addition, testis samples were collected from four different males. Fishes were 4 months old and weighted 0.6 to 0.7 g for females and 0.5 to 0.6 g for males. All the samples were immediately frozen in liquid nitrogen and subsequently stored at −80 °C until RNA extraction.

### RNA extraction

Tissues were homogenized in Tri reagent (Sigma-Aldrich, USA) and total RNA was extracted according to manufacturer’s instructions. RNA quality was checked using an Agilent 2100 Bioanalyzer system. The presence of miRNAs in the samples was assessed using a small RNA chip (Agilent Technologies, USA) and RNA quantity was measured on a Nanodrop 1000 Spectrophotometer (Thermo Scientific, USA).

### Microarray design

We used a microarray platform displaying 3800 distinct miRNAs from different vertebrate species (fish, birds and mammals). The design was performed as previously described using e-array custom platform (Agilent) with miRBase version 19.0 and subsequently deposited in Gene Expression Omnibus (GEO) database under the reference GPL21776^20^. For each target miRNA in miRBase, 16 probes were synthesized that were divided into two to four groups and have limited variations in sequence and size (one or two nucleotide differences at the 3′ and 5′ extremities). This led to robust miRNA expression analysis. All the available vertebrate miRNAs in miRBase version 19.0 (http://www.mirbase.org) were used (*Oryzias latipes, Danio rerio, Fugu rubripes, Tetraodon nigroviridis, Cyprinus corpio, Hippoglossus hipoglossus* and *Paralichthys olivaceus*). The miRNAs from rodents (*Mus musculus* and *Rattus norvegicus*), frogs (*Xenopus laevis* and *Xenopus tropicalis*), humans (*Homo sapiens*), and chickens (*Gallus gallus*) were subsequently added (Supplemental Fig. S5).

### Microarray hybridization, data processing and miRNA annotation

Samples were processed and hybridized on the miRNA 8 × 60 K microarray as previously described, using the miRNA Microarray System with miRNA Complete Labeling and Hyb Kit (Agilent v2.2) according to the manufacturer’s instructions with minor modifications^20^. Total RNA (200 ng) with labeling Spike-In was dephosphorylated for 30 minutes at 37 °C. RNA was denatured with DMSO at 100 °C for 7 minutes. RNA was then labeled by terminal ligation of cyanine3-pCp using T4 ligase for 2 hours at 16 °C. Purification of labeled RNA was done using Micro Bio-Spin column (Bio-Rad) to eliminate free cyanine3-pCp. Hybridization was performed with hybridizations Spike-In at 55 °C, 20 rpm for 20 hours. Slides were washed for 5 minutes with wash buffer 1 at room temperature and wash buffer 2 at 37 °C and immediately scanned. After labeling with cyanine3-pCp and RNA hybridization, slides were scanned with an Agilent Scanner (Agilent DNA Microarray Scanner; Agilent Technologies) using the following settings: profile = AgilentHD_miRNA, channels = Green, resolution = 3 μm, TIFF = 20 bit. Signal intensity was quantified using the Feature Extraction software (Agilent v10.7.3.1). Features with low signal (lower than three times the background level) were excluded. The data were processed using the GeneSpring software (Agilent v.13.0) using GmedianSignal values and subsequently normalized using quantile normalization. MiRNAs for which at least 8 of the 16 probes displayed a significant overexpression in the ovary, with at least 2-fold overabundance in the ovary in comparison to other tissues, were selected for further analysis as previously described^20^. Corresponding data were deposited in Gene Expression Omnibus (GEO) database under the reference GSE80823.

### Genome mapping, RNA folding and homologous primers design

To identify the corresponding medaka miRNAs, the overabundant miRNA sequences were aligned (ENSEMBL BLAST using short sequences setting) on the medaka genome sequence available in ENSEMBL (release 83). Two to 4 hits with the best identity and coverage scores were considered as possible loci for the corresponding medaka miRNA gene(s). The corresponding genomic region of each hit was considered as the mature sequence and used to design the forward medaka homologous primers for QPCR. Each region was subsequently extended to the opposite arm to tentatively obtain the whole precursor sequence. These predicted sequences were then used for *in silico* prediction of the hairpin using the RNAfold web server with default settings^62^. We used −18 kcal/mol as a minimum free energy (MFE) threshold^63^.

### Target prediction

The genes targeted by miR-4785 and miR-6352 were predicted using TargetScan^64^ and miRanda^65^ with default parameters. For TargetScan, we used TargetScan human and TargetScan mouse to predict miR-4785 and miR-6352 targets, respectively. For each miRNA, the 20 first candidates predicted by TargetScan were selected. Among them, genes predicted to be targeted by both algorithms (Targetscan and miRanda) were considered as putative targets.

### Reverse transcription

Total RNA (100 ng) was reverse transcribed in a final volume of 10 μl. The reaction mixture included 1 μl of 10x poly(A) polymerase buffer, 0.1 mM of ATP, 1 μM of RT-primer, 0.1 mM of each deoxynucleotide (dATP, dCTP, dGTP and dTTP), 100 units of SuperScript III reverse transcriptase (Invitrogen, USA) and 1 unit of poly(A) polymerase (New England Biolabs, USA). The reaction was then incubated at 42 °C for 1 hour, followed by enzyme inactivation at 95 °C for 5 minutes. The sequence of the reverse transcription primer was 5′-AAGCAGTGGTATCAACGCAGAGTACTTTTTTTTTTTTTTTTTTTTTTTTTTTTTTVN-3′ (where V is A, C or G and N is A, C, G or T).

### Quantitative PCR

Quantitative PCR (QPCR) was performed in 10 μl reactions containing the reverse transcription (RT) products (dilution 1:50), 100 nM of each primer and 1× SYBR FAST qPCR Master Mix (Fast SYBR Green Master Mix kit; Applied Biosystems) as indicated in the manufacturer’s instructions. QPCR was performed on a StepOnePlus thermocycler (Applied Biosystems) using the following program: (1) 95 °C for 3 minutes for the initial denaturation, (2) 40 cycles of 95 °C for 3 seconds, and (3) 60 °C for 30 seconds. The sequence of the universal reverse primer was 5′-AAGCAGTGGTATCAACGCAGAG-3′. The sequences of the forward heterologous primers corresponded to the miRNA sequences present on the microarray (Supplemental Fig. S2). The sequences of the forward homologous primers corresponded to the genomic sequences identified on the medaka genome (Supplemental Fig. S4). The relative abundance of the target cDNA within a sample set was calculated from serially diluting the cDNA pool using Applied Biosystems StepOne software. Before analysis, miRNA expression was normalized to the reference gene 18 S^66^.

### Statistical analyses

ANOVA was performed to detect differential expression between the different tissues in the microarray experiments. These analyses were followed by Benjamin-Hochberg correction. For QPCR analysis, we used p < 0.05 as a threshold and the Wilcoxon test for indicating significant differences between samples.

## Additional Information

**Accession codes**: The array design (platform # GPL21776) and microarray expression data (accession # GSE80823) were deposited in the gene expression omnibus (GEO) database (http://www.ncbi.nlm. nih.gov/geo/).

**How to cite this article**: Bouchareb, A. *et al*. Genome-wide identification of novel ovarian-predominant miRNAs: new insights from the medaka (*Oryzias latipes*). *Sci. Rep.* **7**, 40241; doi: 10.1038/srep40241 (2017).

**Publisher's note:** Springer Nature remains neutral with regard to jurisdictional claims in published maps and institutional affiliations.

## Supplementary Material

Supplemental Figures

## Figures and Tables

**Table 1:** Characterization of a subset of novel medaka miRNAs predominantly expressed in the ovary.

miRNA	QPCR heterologous	Locus	Sequence	Location	Folding	QPCR homologous	miRNA origins
miR-6352-5p	+	1	CAGGGAACAGGACCCCAGCT	9:31095668–31095687	+	+	miRtron/ENSORLG00000017404
		2	AACTGGGAAAAGGACCCCAG	3:26171679–26171698	−	+	miRtron/*myo5aa*
		3	TTTGGGAAAAAGATCCCAGCT	1:31427215–31427235	−	−	NC
		4	AAATGGGAAAAGCACCCCAG	20:10904125–10904144	−	−	NC
miR-4785–3p	+	1	GAGTCTGCGTCGCCGCCAGC	16:16874299–16874318	+	+	miR/*rasip*
		2	TGATCTCGGAGACGCCGCCAGC	scaffold983:45388–45409	+	+	miR
		3	CGAGTCGGCGATGCCGCCGCC	9:761851–761871	−	−	NC
miR-878–3p	+	1	GCATGACAACATACTGGGTA	12:12844102–12844121	−	+	miR
		2	GCATGAAACCATACTGGGTC	13:25685522–25685541	−	−	NC
miR-1288-3p	+	1	TGGACTGTCCTGATGTGGAGA	6:5567815–5567835	−	−	NC
		2	TGGACAGCCCTGATGTGGAG	1:27438143–27438162	+	+	miR
miR-4653-3p	+	1	GAGTTAAGGGTAGTTTGGAGA	18:4398355–4398375	−	+	miR
		2	CTCTGTTAAGGGTTGCCTGGA	ultracontig49:380302–380322	−	−	NC
		3	AGAAAGTTGAGGGTTGCTTGG	21:27820909–27820929	−	−	NC
miR-729–3p	+	1	CATGGGTATGAAACGACCCGGGT	20:6728838–6728860	−	−	NC
		2	CATGGGTATGATACGACCTCA	scaffold1021:47363–47383	+	+	miR
		3	GGTATGATTCGGCCTGGGTT	15:3091445–3091464	−	−	NC
miR-487b-3p	+	1	CGGCGTACAGGGTCATCCAC	22:3962447–3962466	−	−	NC
		2	GTCCCCTACAAGGTCATCCACTT	10:7430304–7430326	−	+	miRtron/*mrpl11*
miR-202-3p	+		AGAGGCATAAGGCATGGGAA	15:1382691–1382710	+	+	miR
miR-743a-3p	−	1	GAAAGACGCCAAAGTGGGT	2:28436804–28436822	−	+	miR
		2	GAAAGTCGCCAAACTGGGT	18:15585090–15585108	−	+	miR
miR-1305-3p	−	1	TTTTCAACTCTATTGGGAAA	12:19213569–19213588	−	−	NC
		2	ATGTTCAACTCTAATGTGAGA	24:18349479–18349499	−	−	NC
		3	TTTTCACCTCTAATGGGATAG	16:14807294–14807314	+	−	miRtron/*fam171a1*
miR-4716-3p	−		CTGGGGGAAGCAAACATGGAGA	7:23549695–23549716	+	−	miR
miR-6131-3p	−	1	CGCTGATCAGATGGGAGTCG	15:2799945–2799964	−	−	NC
		2	ACTGGTGAGATGGGAGTGGC	12:21774089–21774108	+	−	miR
miR-10d-3p	−		TACCCTGTAGAACCGAATGTGT	15:4353766–4353787	+	−	miR
miR-6717-5p	−	1	GGCGATGGGGGGATGCAGAGA	19:8376541–8376561	+	−	miRtron/*shank1*
		2	AAAGATGTGGGGATGTGGAGA	scaffold1101:27027–27047	−	−	NC
miR-345-3p	−	1	CCCTGAACTCGGGGTATGGA	16:8875414–8875433	−	−	NC
		2	CTGAACTAGGGTTCAGGAGA	16:14238722–14238741	−	−	NC
miR-697-3p	−	1	AGCATCCTGTTCCTGTGGAG	scaffold5397:869–888	−	−	NC
		2	ACATCCTGGACCTGTGGA	scaffold692:99305–99322	−	−	NC
miR-3198-3p	−	1	GTGGAGTCCTGAGGACTGGAG	5:11964002–11964022	−	−	NC
		2	GTGGAGTCCTGCAGAATGGAG	19:5590938–5590958	−	−	NC
miR-4713-3p	−	1	TGGGATCCAGACAGAGGGAGAA	15:20189222–20189243	−	−	NC
		2	TGGGATGCAGAAAGTGGGAGAA	17:30648359–30648380	−	−	NC
		3	AAAGATCCAGACAGTTGGAGA	scaffold693:106481–106501	−	−	NC
miR-5581-5p	−	1	AGCCTTCCAGGAAAAATGGAG	scaffold4519:2360–2380	−	−	NC
		2	AGCCTTCCAGGAAAAATGGACA	scaffold5870:432–453	−	−	NC
miR-892b-3p	−	1	ACTGGGTCCTTTCTGTGTAGA	3:12292877–12292897	−	−	NC
		2	CACTGGCTCTTTTCTGGGAA	23:15587446–15587465	−	−	NC

Eight miRNAs displayed an ovarian-predominant expression profile when using heterologous and homologous QPCR primers. In addition, *in silico* folding predictions allowed characterizing 13 loci that encode 11 different miRNAs. +, successful QPCR or hairpin structure prediction. NC, not confirmed.
