# Supplementary material for: Genome-wide identification of novel ovarian-predominant miRNAs: new insights from the medaka (Oryzias latipes)
Source: Sci Rep. 2017 Jan 10;7:40241. doi: 10.1038/srep40241 (PMC5223123; doi:10.1038/srep40241)
Supplement: Supplemental Figures [file srep40241-s1.pdf]

Genome-wide identification of novel ovarian-predominant miRNAs: new insights from the medaka (*Oryzias latipes*)

Amine Bouchareb, Aurélie Le Cam, Jérôme Montfort, Stéphanie Gay, Thaovi Nguyen, Julien Bobe*, Violette Thermes*

LPGP, INRA, 35000 Rennes, France

* Equal contribution

Correspondence to: Violette Thermes, violette.thermes@inra.fr

Supplemental Fig. S1: Ovarian miRNAs over-abundance. Fold change of miRNA expression in ovary compared to the mean of other tissues or to the mean of testis.

Supplemental Fig. S2: Heterologous miRNA primer sequence.

Supplemental Fig. S3: Predicted miRNA hairpin structures.

Supplemental Fig. S4: Homologous miRNA primer sequences.

Supplemental Fig. S5: Generic vertebrate miRNAs 8x60K microarray design.

Supplemental Fig. S6: Predicted target genes for miR4785 and miR6352.

Supplemental Fig. S1: Ovarian miRNAs over-abundance. Fold change of miRNA expression in ovary compared to the mean of other tissues or to the mean of testis.

Microarray probe	Fold-change ovary *vs* tissues	Fold-change ovary *vs* testis
hsa-miR-4785	187.82	134.21
mmu-miR-6352	59.88	46.81
hsa-miR-1305	31.23	26.91
hsa-miR-4713-3p	28.66	25.15
hsa-miR-4716-3p	23.54	20.02
rno-miR-202-3p	23.18	19.7
mmu-miR-202-3p	21.97	22.66
dre-miR-202-3p	11.13	1.78
hsa-miR-6717-5p	22.28	18.69
hsa-miR-5581-5p	21.97	15.21
mmu-miR-697	21.3	19.49
hsa-miR-3198	19.88	16.85
hsa-miR-6131	19.7	17.28
hsa-miR-4653-3p	18.99	15.78
hsa-miR-487b-3p	15.11	14.22
hsa-miR-5003-5p	15.11	6.83
hsa-miR-1288-3p	15.03	14.23
hsa-miR-3138	14.78	13.99
hsa-miR-3125	13.17	15.24
dre-miR-729	12.77	10.74
rno-miR-743a-3p	12.63	11.41
rno-miR-878	12.26	11.31
hsa-miR-892b	11.89	11.18
rno-miR-345-3p	11.73	11.23
rno-miR-770-3p	11.55	10.85
mmu-miR-878-3p	11.04	10.34
rno-miR-3561-5p	9.59	8.73
mmu-miR-1231-5p	9.52	9.99
hsa-miR-500a-5p	9.48	8.28
dre-miR-10d-3p	8.67	7.39
rno-miR-150-3p	7.58	8.43
rno-miR-501-5p	7.11	6.71
hsa-miR-6512-5p	6.96	6.44
mmu-miR-3070-3p	6.95	6.28
mmu-miR-3058-5p	6.74	6.58
hsa-miR-1226-5p	6.67	4.51
hsa-miR-3127-5p	6.58	6.71
mmu-miR-3070-2	6.33	5.07
rno-miR-3562	6.16	4.4
mmu-miR-128-2	5.24	5.87
rno-miR-547-3p	4.83	4.79
hsa-miR-5194	4.54	3.58
rno-miR-1912-3p	4.44	4.28
mmu-miR-202-5p	4.11	0.56
rno-miR-202-5p	1.59	0.74
hsa-miR-575	4.02	3.77
gga-miR-1577	4.01	3.93
hsa-miR-6124	4	6.32
ola-miR-187	3.93	3.72
hsa-miR-4518	3.88	3.8
mmu-miR-470-3p	3.75	3.67
hsa-miR-1295a	3.55	3.48
hsa-miR-320a	3.42	3.84
mmu-miR-6412	3.38	2.74
rno-miR-6326	3.25	3.02
rno-miR-501-5p	2.91	2.93
hsa-miR-4535	2.86	2.8
hsa-miR-135a-3p	2.83	2.01
mmu-miR-330-3p	2.79	2.79
hsa-miR-4648	2.73	2.73
dre-miR-31	2.67	2.87
hsa-miR-513c-3p	2.65	2.7
rno-miR-3065-3p	2.61	2.59
hsa-miR-6134	2.58	2.27
hsa-miR-4458	2.52	2.66
hsa-miR-5196-5p	2.48	2.22
hsa-miR-145-3p	2.43	0.51
mmu-miR-1971	2.41	2.09
mmu-miR-3104-5p	2.28	2.01
mmu-miR-6405	2.25	2.14
gga-miR-1466	2.18	2.46

Supplemental Fig. S2: Heterologous miRNA primer sequences.

miRNA	Primer sequence
dre-miR-202-3p	AGAGGCAUAGGGCAUGGGAAAA
dre-miR-29	AUGGGUAUGAUACGACCUGGGUU
hsa-miR-1288-3p	UGGACUGCCCUGAUCUGGAGA
hsa-miR-4653-3p	UGGAGUUAAGGGUUGCUUGGAGA
hsa-miR-4785	AGAGUCGGCGACGCCGCCAGC
hsa-miR-487b-3p	AAUCGUACAGGGUCAUCCACUU
mmu-miR-6352	UUUGGGAAAAGGACCCCAGCUC
rno-miR-878	GCAUGACACCAUACUGGGUAGA
rno-miR-743a-3p	GAAAGACGCCAAACUGGGUAGA
hsa-miR-1305-3p	UUUUCAACUCUAAUGGGAGAGA
hsa-miR-4716-3p	AAGGGGGAAGGAAACAUGGAGA
hsa-miR-6131-3p	GGCUGGUCAGAUGGGAGUG
dre-miR-10d-3p	CAGAUUCGGUUUUAGGGGAGUA
hsa-miR-6717-5p	AGGCGAUGUGGGGAUGUAGAGA
rno-miR-345-3p	CCCUGAACUAGGGGUCUGGAGA
mmu-miR-697-3p	AACAUCCUGGUCCUGUGGAGA
hsa-miR-3198-3p	GUGGAGUCCUGGGGAAUGGAGA
hsa-miR-4713-3p	UGGGAUCCAGACAGUGGGAGAA
hsa-miR-5581-5p	AGCCUUCCAGGAGAAAUGGAGA
hsa-miR-892b-3p	CACUGGCUCCUUUCUGGGUAGA

Supplemental Fig. S3: Predicted miRNA hairpin structures.

miRNA name	Medaka genomic locus	RNA Folding	Lenght (nt)	MFE (-kcal/mol)
mir-6352a	9:31095668-31095687	CAGGUUGGGGGUCAGGGAACAGGACCCCAGCUAAAGGGAGUCUUCGGUUCUGUCAAAACCAGAGAACCAACAGCAGAAC<br>...(((((...((.((..((((((((..((((......)).))..)))))))).....)).))...))))))........	80	21.80
mir-4785a	16:16874299-16874318	CCAGAGGAGUGCGAGCGUGGACACCGUGGAAGAAACACAGAGCUUCGGAGGAGCAUCAGUGACAUGAACCUGAGUCUGCG<br>.....((...((((.(((((((..((.((......(((.(((((((...))))).)).)))......)))).)))))))<br>UCGCCGCCAGCAAAACCAU<br>))))..))...........	80	24.40
mir-4785b	scaffold983:45388-45409	CCCUCCGUUUUGAUCUGGGUGAGUCUCAGCACCAGCUCUUGGAGAGCAUCGACCAGCUCAUCUGUGAUCUCGGAGACGCC<br>..(((((....(((((((((((((..........(((((...)))))........))))))))).)))).))))).....<br>GCCAGCCCG<br>.........	80	25
mir-729	scaffold1021:47363-47383	CCCUCCAGAGCAGGGGCUGUAUCAUAACCAGGCUGUGAGCUGAACGAGCAUGGGUAUGAUACGACCUCAGUU<br>........(((.((((.(((((((((.(((.(((((.......)).))).))).))))))))).)))).)))	72	25.2
mir-1288	1:27438143-27438162	UGCAGUGAGGGCUGGAAAACAGACAGGGUGUGGAUCAUAUGGAUAGCAAUGUUGGACAGCCCUGAUGUGGAGCUUUGG<br>.(((...(((((((...((((......(((((...)))))..........))))...)))))))..)))..........	78	19.9
mir-4716	7:23549695-23549716	UGAUUACAGAAGUGUUUAAGGUUUCCUCUAUGCGAAGAUGAGUGUUUGUGUUCAUCUAUUGUGAACUGGGGGAAGCAAAC<br>.......(((((((((...((((((((((((((((((((((........))))))).)))))...)))))))))))))<br>AUGGAGAUUCUUUCAU<br>)).....)))).....	80	23.8
mir-6717	19:8376541-8376561	GACAUGGCGAUGGGGGGAUGCAGAGAGAGAGAUUUCUCUCUCCCUCUCUUCUUCGUGGCCAUCACUGGG<br>...(((((.(((((((((....(((((((((....)))))))))....)).))))))).))))).......	71	31.4
mir-6131	12:21774089-21774108	CAGGUAAAUGCAGGUCACCCUCAUCUUAACCCCAACAUGUAGAAACUGUUGCAUACUGGUGAGAUGGGAGUGGCUGGUGU<br>.......((((.((((((.(((((((((...(((..((((((......))))))..)))))))))))).)))))).))))<br>AAC<br>...	80	27.9
mir-1305	16:14807294-14807314	GAAGGUCUUCUGAUGAAACAAAAUGUUCUGCUUUGUUUGGAAUUUUUAUUUUGAGCAAAGUACACAUCUUUUCACCUCUA	80	19.1

		.(((((..(((.((((((....((((..(((((((((((((((....))))))))))))))).)))).)))).......<br>AUGGGAUAGCUCUUAAAC<br>)).)))..)).))))....		
mir-202	15:1382691-1382710	CUCGCUGUUCCUUUUUCCUAUGCAUAUACUUCUUUGACAUGUAACUUUAAAGAGGCAUAAGGCAUGGGAAAAUGGGGCUG<br>(((((.(((((.(((((((((((.(((.(((((((((.........))))))))).)))..))))))))))).))))).)<br>CAGAG<br>).)))	80	35.6
mir-10d	15:4353766-4353787	GCCGUGACGGGAACCUUCGCCCAUACAUACCCUGUAGAACCGAAUGUGUGUGGAGCUGCCUCAGUCACAGAUUGGGUUCU<br>.....((.((......(((((((((...(((((.(((((((.(((.((((..(((....)))...)))).))).))))))<br>AGGGGAGUCUAUGGGCGAUGACUAACCAUCUAUCA<br>).))).)).)))))))))).......)).)).....	80	47.1

Supplemental Fig. S4: Homologous miRNA primer sequences.

miRNA	Primer sequences
miR-1288	TGGACTGTCCTGATGTGGAGA
miR-202-3p	AGAGGCATAAGGCATGGGAA
miR-4653	GAGTTAAGGGTAGTTTGGAGA
miR-4785a	GAGTCTGCGTCGCCGCCAGC
miR-4785b	TGATCTCGGAGACGCCGCCAGC
miR-487b	GTCCCCTACAAGGTCATCCACTT
miR-6352a	CAGGGAACAGGACCCCAGCT
miR-6352b	AACTGGGAAAAGGACCCCAG
miR-729	CATGGGTATGATACGACCTC
miR-743aa	GAAAGACGCCAAAGTGGGT
miR-743ab	GAAAGTCGCCAAACTGGGT
miR-878	GCATGACAACATACTGGGTA
miR-6352-3	TTTGGGAAAAAGATCCCAGCT
miR-6352-4	AAATGGGAAAAGCACCCCAG
miR-4785-3	CGAGTCGGCGATGCCGCCGCC
miR-878-2	GCATGAAACCATACTGGGTC
miR-1288-3p-2	TGGACAGCCCTGATGTGGAG
miR-4653-3p-2	CTCTGTTAAGGGTTGCCTGGA
miR-4653-3p-3	AGAAAGTTGAGGGTTGCTTGG
miR-729-3p-2	CATGGGTATGAAACGACCCGGGT
miR-729-3p-3	GGTATGATTCGGCCTGGGTT
miR-487b-3p-2	CGGCGTACAGGGTCATCCAC
miR-1305-3p-1	TTTTCAACTCTATTGGGAAA
miR-1305-3p-2	ATGTTCAACTCTAATGTGAGA
miR-1305-3p-3	TTTTCACCTCTAATGGGATAG
miR-4716-3p	CTGGGGGAAGCAAACATGGAGA
miR-6131-3p-1	CGCTGATCAGATGGGAGTCG
miR-6131-3p-2	ACTGGTGAGATGGGAGTGGC
miR-10d-3p	TACCCTGTAGAACCGAATGTGT
miR-6717-5p-1	GGCGATGGGGGGATGCAGAGA
miR-6717-5p-2	AAAGATGTGGGGATGTGGAGA
miR-345-3p-1	CCCTGAACTCGGGGTATGGA
miR-345-3p-2	CTGAACTAGGGTTCAGGAGA
miR-697-3p-1	AGCATCCTGTTCCTGTGGAG
miR-697-3p-2	ACATCCTGGACCTGTGGA
miR-3198-3p-1	GTGGAGTCCTGAGGACTGGAG
miR-3198-3p-2	GTGGAGTCCTGCAGAATGGAG
miR-4713-3p-1	TGGGATCCAGACAGAGGGAGAA
miR-4713-3p-2	TGGGATGCAGAAAGTGGGAGAA
miR-4713-3p-3	AAAGATCCAGACAGTTGGAGA
miR-5581-5p-1	AGCCTTCCAGGAAAAATGGAG
miR-5581-5p-2	AGCCTTCCAGGAAAAATGGACA
miR-892b-3p-1	ACTGGGTCCTTTCTGTGTAGA
miR-892b-3p-2	CACTGGCTCTTTTCTGGGAA

Supplemental Fig. S5: Generic vertebrate miRNAs 8x60K microarray design.

Rank	Species	# of miRNAs per species in miRBase V19	# of miRNAs present on the array
1	*Oryzias latipes*	144 (304 probes)	144
2	*Danio rerio*	245 (519 probes)	228
3	*Fugu rubripes*	108 (224 probes)	10
4	*Tetraodon nigroviridis*	109 (226 pobes)	3
5	*Cyprinus corpio*	146 (314 probes)	68
6	*Hippoglossus hipoglossus*	1 (3 probes)	1
7	*Paralichthys olivaceus*	37 (82 probes)	19
8	*Mus musculus*	1247 (3105 probes)	1159
9	*Rattus norvegious*	719 (1730 probes)	374
10	*Xenopus tropicalis*	173 (369 probes)	72
11	*Xenopus laevis*	21 (47 probes)	2
12	*Homo sapiens*	2006 (4774 probes)	1665
13	*Gallus gallus*	789 (1662 probes)	55

Supplemental Fig. S6: Predicted target genes for miR-4785 and miR-6352.

MIR4785		MIR6352	
ENSG00000244045	TMEM199	ENSMUSG00000020495	Smg8
ENSG00000196152	ZNF79	ENSMUSG00000036854	Hspb6
ENSG00000174521	TTC9B	ENSMUSG00000013353	4931406B18Rik
ENSG00000141574	SECTM1	ENSMUSG00000033943	Mga
ENSG00000167670	CHAF1A	ENSMUSG00000043832	Clec4a3
ENSG00000197483	ZNF628	ENSMUSG00000050558	Prokr2
ENSG00000205927	OLIG2	ENSMUSG00000089774	Slc5a3
ENSG00000125788	DEFB126	ENSMUSG00000074365	Crxos
ENSG00000112679	DUSP22	ENSMUSG00000070695	Cntnap5a
ENSG00000170820	FSHR	ENSMUSG00000038893	Fam117a
ENSG00000179133	C10orf67	ENSMUSG00000027905	Ddx20
		ENSMUSG00000032097	Ddx6
		ENSMUSG00000035456	Prdm8
		ENSMUSG00000094958	3110021N24Rik
		ENSMUSG00000059060	Rad51b
		ENSMUSG00000062157	Ifnlr1
		ENSMUSG00000020463	Smek2

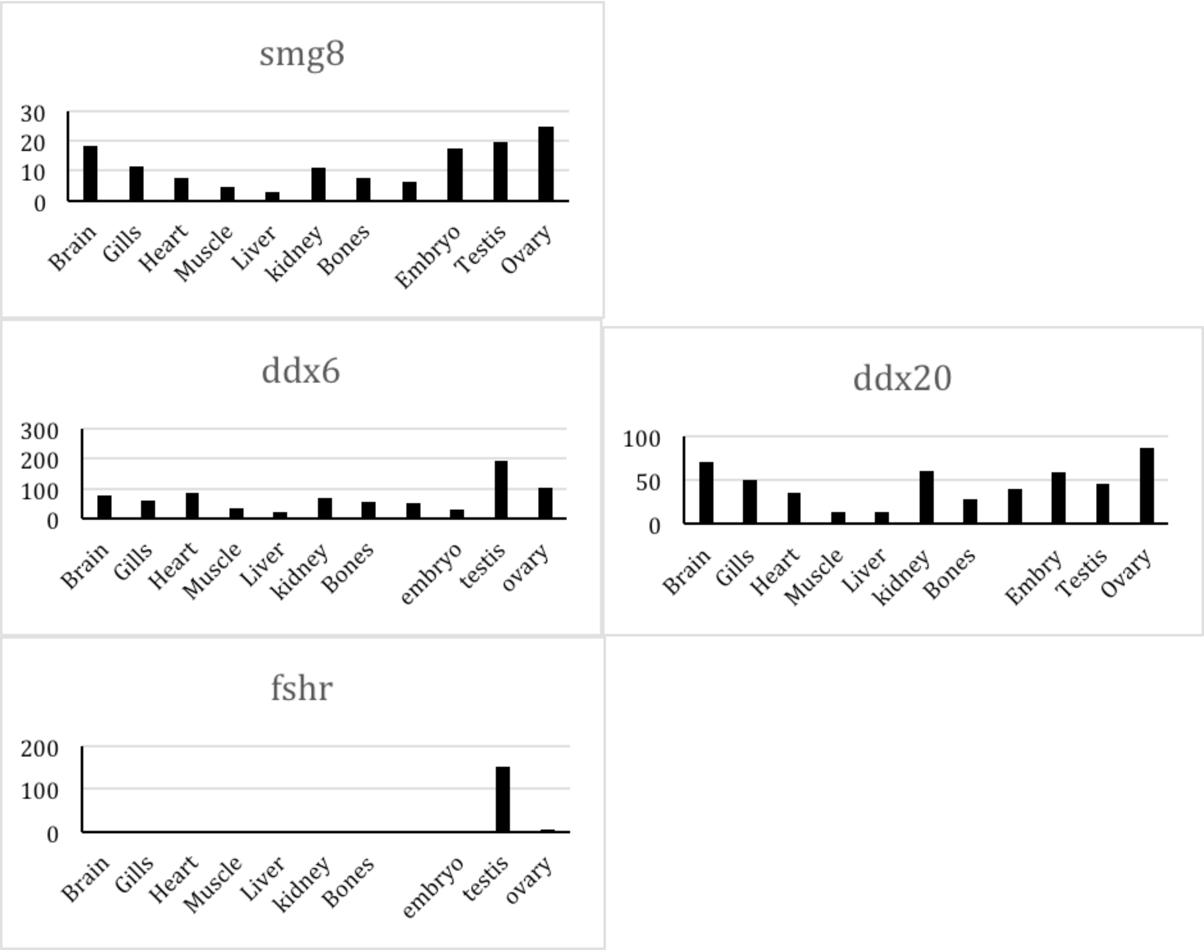

smg8

Tissue	Mean
Brain	18,41
Gills	11,34
Heart	7,78
Muscle	4,58
Liver	2,67
kidney	11,11
Bones	7,48
Intestine	6,3
Embryo	17,54
Testis	19,82
Ovary	24,61

ddx20

Tissue	Mean
Brain	70,12
Gills	50,84
Heart	36,43
Muscle	14,18
Liver	13,17
kidney	60,17
Bones	27,99
Intestine	39,86
Embryo	59,39
Testis	46,48
Ovary	86,35

ddx6

Tissue	Mean
Brain	75,73
Gills	60,96
Heart	84,43
Muscle	33,29
Liver	22,5
kidney	69,73
Bones	56,84
Intestine	49,37
embryo	30,54
testis	191,66
ovary	102,45

fshr

Tissue	Mean
Brain	2,18
Gills	0,06
Heart	0,25
Muscle	0
Liver	0
kidney	0,06
Bones	0,06
Intestine	0
embryo	0
testis	153,2
ovary	5,39
